# Lipid metabolism-related gene expression in the immune microenvironment predicts prognostic outcomes in renal cell carcinoma

**DOI:** 10.3389/fimmu.2023.1324205

**Published:** 2023-11-27

**Authors:** Qian Zhang, Bingbiao Lin, Huikun Chen, Yinyan Ye, Yijie Huang, Zhen Chen, Jun Li

**Affiliations:** ^1^ Department of Rehabilitation Medicine, Seventh Affiliated Hospital, Sun Yat-sen University, Shenzhen, Guangdong, China; ^2^ Department of Urology, Seventh Affiliated Hospital, Sun Yat-sen University, Shenzhen, Guangdong, China; ^3^ Department of Radiotherapy, Cancer Hospital of Shantou University Medical College, Shantou, Guangdong, China

**Keywords:** renal cell carcinoma, lipid metabolism, immune infiltration, risk model, biomarker

## Abstract

**Background:**

Rates of renal cell carcinoma (RCC) occurrence and mortality are steadily rising. In an effort to address this issue, the present bioinformatics study was developed with the goal of identifying major lipid metabolism biomarkers and immune infiltration characteristics associated with RCC cases.

**Methods:**

The Cancer Genome Atlas (TCGA) and E-MTAB-1980 were used to obtain matched clinical and RNA expression data from patients diagnosed with RCC. A LASSO algorithm and multivariate Cox regression analyses were employed to design a prognostic risk model for these patients. The tumor immune microenvironment (TIME) in RCC patients was further interrogated through ESTIMATE, TIMER, and single-cell gene set enrichment analysis (ssGSEA) analyses. Gene Ontology (GO), KEGG, and GSEA enrichment approaches were further employed to gauge the mechanistic basis for the observed results. Differences in gene expression and associated functional changes were then validated through appropriate molecular biology assays.

**Results:**

Through the approach detailed above, a risk model based on 8 genes associated with RCC patient overall survival and lipid metabolism was ultimately identified that was capable of aiding in the diagnosis of this cancer type. Poorer prognostic outcomes in the analyzed RCC patients were associated with higher immune scores, lower levels of tumor purity, greater immune cell infiltration, and higher relative immune status. In GO and KEGG enrichment analyses, genes that were differentially expressed between risk groups were primarily related to the immune response and substance metabolism. GSEA analyses additionally revealed that the most enriched factors in the high-risk group included the stable internal environment, peroxisomes, and fatty acid metabolism. Subsequent experimental validation *in vitro* and *in vivo* revealed that the most significantly differentially expressed gene identified herein, ALOX5, was capable of suppressing RCC tumor cell proliferation, invasivity, and migration.

**Conclusion:**

In summary, a risk model was successfully established that was significantly related to RCC patient prognosis and TIME composition, offering a robust foundation for the development of novel targeted therapeutic agents and individualized treatment regimens. In both immunoassays and functional analyses, dysregulated lipid metabolism was associated with aberrant immunological activity and the reprogramming of fatty acid metabolic activity, contributing to poorer outcomes.

## Introduction

Renal cell carcinoma (RCC) incidence and mortality rates are continually rising, comprising ~90% of all malignant renal tumors and 3% of all malignancies (1, 2). An estimated 60% of RCC cases are asymptomatic such that they are only incidentally discovered during physical examinations (3), with patients only rarely exhibiting a triad of characteristic symptoms including an abdominal mass, gross hematuria, and flank pain (4). While ablative or surgical approaches can successfully treat ccRCC in many patients, up to one in three already harbor distant metastases when first diagnosed, and these patients face a poor prognosis (5–8). As such, researchers have increasingly sought to enable early-stage detection of RCC and to design new approaches to assessing the prognosis of affected patients.

Research has recently demonstrated that genetic testing technologies can enable the more reliable early-stage detection of RCC. As a result, there have been many analyses focused on the identification of novel prognostic and/or diagnostic biomarkers associated with this cancer type, providing new opportunities to improve the identification of this form of cancer and prolong patient survival (9, 10). Kim et al. explored the clinical relevance of nine biomarkers associated with disease progression and other pathophysiological changes in RCC and ultimately determined that both pS6 and Ki-67 were predictive of primary RCC patient prognosis (11). An et al. determined that a nomogram constructed based on TNM staging and higher levels of the independent prognostic factor CXCR4 was capable of predicting clinical outcomes in ccRCC patients (12). While these results are extremely promising, they are not exhaustive and there remain many opportunities to define novel diagnostic and prognostic genes associated with this cancer type. The systematic characterization of these biomarkers may yield new mechanistic insight into the pathogenesis of RCC while also supporting the design of new diagnostic and treatment strategies that can be implemented in a clinical setting.

As a canonical hallmark of cancer, metabolic reprogramming can allow cancer cells to modulate their lipid metabolism such that they can thrive even under conditions of nutrient deprivation by ensuring that cell signaling, energy storage, and membrane biosynthesis can persist (13). ccRCC cases are so named because the high levels of lipids and glycogen that accumulate within these tumor cells via lipid uptake, synthesis, and storage in lipid droplets cause them to appear transparent when visualized with a microscope. When kidney damage occurs, renal epithelial cells undergo metabolic pathway alterations that boost lipid accumulation (14). Innovative therapeutic methods for regulating the lipid balance of cancer cells, whether by inhibiting biosynthesis or uptake of fatty acids and cholesterol, have demonstrated promising outcomes in both *in vitro* and *in vivo* studies (14). Despite this important characteristic, research focused on metabolic remodeling in ccRCC has not received as much focus as in other cancer types to date.

The tumor immune microenvironment (TIME) is a niche wherein tumor cells engage in complex interactions with the stroma, the extracellular matrix (ECM), and a range of immune cells including T cells, B cells, natural killer (NK) cells, and macrophages. While many immune cells can initially target and eliminate emergent tumor cells early in the oncogenic process, over time tumors develop approaches to suppressing the cytotoxic effects of these immune cells and/or evading immune-mediated detection. The composition of the TIME includes a range of cytokines, chemokines, and immune checkpoint molecules that shape tumor growth, progression, and responses to particular treatments (15–17). In the case of advanced RCC, there is a growing trend of recommending and researching molecular-targeted drugs, specifically tyrosine kinase inhibitors (TKIs), and immune checkpoint inhibitors (ICIs). Owing to the presence of a dynamic, adaptive, and heterogeneous TIME, along with the unique glucose and lipid metabolism in RCC, this cancer can exhibit diverse forms of resistance to TKIs and ICIs. Therefore, comprehensive research on the TIME is essential for advancing the development of cancer immunotherapies (18).

Here, lipid metabolism- and overall survival (OS)-related differentially expressed genes (DEGs) were analyzed in a comprehensive manner in an effort to more fully understand the association between lipid metabolism, TIME composition, and survival outcomes in ccRCC patients. A DEG-based risk model was then designed to examine the prognostic relevance of these lipid metabolism- and OS-related DEGs in ccRCC. Together, these findings have the potential to offer new insights into the mechanisms governing ccRCC development and progression, thus providing a foundation for efforts to more reliably diagnose and treat this disease in a targeted manner on an individualized basis.

## Materials and methods

### Data collection

RNA-seq data and associated clinical information were obtained from The Cancer Genome Atlas (TCGA) database (https://portal.gdc.cancer.gov/) and E-MTAB-1980 (https://www.ebi.ac.uk/), which were utilized as a training cohort and an independent validation cohort in this study respectively. To be eligible for inclusion, samples had to be diagnosed ccRCC cases with available gene expression and clinical data, including information related to patient age, sex, clinical stage, pathological grade, survival status, and survival duration. Samples were excluded if clinical information or gene expression data were incomplete. In total, 526 ccRCC samples from TCGA and 101 ccRCC samples from the EMBL-EBI database meeting these inclusion criteria were incorporated into the present analyses. Lipid metabolism-related genes (N=742) were identified using the Molecular Signature Database (MSigDB) (19).

### Identification of lipid metabolism and survival-related differentially expressed genes

To explore differences in lipid metabolism-related gene expression and their association with OS in ccRCC, the R ‘limma’ package was used to identify DEGs when comparing control and ccRCC tumor tissue samples (20). DEGs were defined as targets that exhibited an adjusted p value (q value) < 0.05 and a |log2FC| >1 (upregulated DEGs) or log2FC <-1 (downregulated DEGs) (21). These genes were presented with Volcano plots and heat maps. The association between genes and ccRCC patient prognosis was evaluated through univariate Cox regression analyses performed with the R ‘survival’ package (22). DEGs associated with both lipid metabolism and OS were identified based on the overlapping DEGs identified when comparing control and diseased samples for lipid metabolism and OS-related gene sets, as visualized using Venn diagrams.

### Risk model establishment, verification, and prediction

The 526 ccRCC samples derived from the TCGA database were separated randomly at a 2:1 ratio into training and validation cohorts. To reduce the number of lipid metabolism and OS-related DEGs included in these analyses, a least absolute shrinkage and selection operator (LASSO) analysis was conducted with the R “glmnet” package, enabling the selection of an optimal minimum lambda value (23). Multivariate Cox regression analyses were employed to select DEGs for the establishment of a risk model, and risk scores were then calculated for individual patients in each cohort with the following formula: Risk Score = expression of Gene1 × coef + expression of Gene2 × coef + expression of Gene3 × coef … Risk score values were then used to stratify patients into low- and high-risk groups. Model predictive efficiency was assessed based on receiver operating characteristic (ROC) curves and the Martingale residuals method. In order to more reliably predict ccRCC patient outcomes, a nomogram incorporating risk scores and clinical features was additionally established (24).

### Immune analyses

Four immune-related algorithms were employed to assess the immune landscape disparities between the high- and low-risk groups. Stromal, immune, and ESTIMATE scores, along with tumor purity were calculated using the ESTIMATE algorithm based on the ratio of immune and stromal cells. Additionally, the TIMER database and CIBERSORT algorithm were also utilized to predict the composition of infiltrating immune cells in each tumour sample. To better understand the immunological characteristics of these samples, 22 infiltrating immune cell types and 29 immune-associated gene sets covering a range of cell types and pathways from MSigDB were analyzed (25). Levels of cell, functional, or pathway enrichment in tumor samples were assessed through a single-sample gene set enrichment analysis (ssGSEA) approach (26).

### Functional enrichment analyses

Gene Ontology (GO) functional enrichment analyses enable the detection of enriched biological terms associated with particular gene sets (27). KEGG pathway enrichment analyses similarly allow for the identification of enriched pathways associated with these genes (28). For this study, GO and KEGG enrichment analyses were performed with the R clusterProfiler package, with the resultant data being visualized with Metascape (29). A false discovery rate-corrected P < 0.05 serves as the cut-off for statistical significance.

GSEA approaches enable the computational evaluation of changes in particular biological activities and pathways in a given dataset. Here, the GSEA program was used to analyze ccRCC patient gene expression profiles using the “c2.cp.kegg.v2023.1.Hs.symbols.gmt” gene set from MSigDB. P < 0.05 served as the cut-off for statistical significance.

### Cell culture

Human ccRCC cell lines from the ATCC cell databases were cultured in DMEM or RPMI-1640 (HyClone, UT, USA) containing fetal bovine serum (FBS; Gemini, CA, USA) and penicillin/streptomycin (Sigma-Aldrich, MO, USA) in a humidified 5% CO_2_ incubator at 37°C. Media was routinely replaced every 2-3 days, and cells were subcultured when confluent by rinsing cells with FBS-free media, detaching them with trypsin (HyClone, UT, USA), resuspending them in complete media, and transferring them to a new dish at a lower culture density.

### qPCR validation

qPCR approaches are widely used to assess gene expression. Relative gene expression in this study was assessed via qPCR using the 2^-ΔΔCt^ method, with β-actin serving as a normalization control (30).

### Western blotting

Western blotting enables the semi-quantitative assessment of protein levels in a given sample. To confirm the results of bioinformatics analyses in the present study, Western blotting was conducted. Initially, RIPA buffer was used to lyse ccRCC cells, and protein lysates were separated via SDS-PAGE, transferred to PVDF membranes (Bio-Rad, CA, USA), and these blots were probed overnight with 1:1000 dilutions of primary antibodies at 4°C. Primary antibodies were anti-ALOX5(Abcam™, ab169755), anti-DPEP1(Abcam™, ab230977), anti-HADH(Abcam™, ab110284), anti-PLIN2(Abcam™, ab108323), anti-SCD5(A13127, ABclonal, Wuhan, China), anti-SLC44A4(A10435, ABclonal, Wuhan, China), anti-TRIB3(Abcam™, ab75846), and anti-UGT8(A16442, ABclonal, Wuhan, China). Membranes were then probed using an HRP-conjugated secondary antibody (ABclonal, A3610, Wuhan, China), followed by protein band detection with a hypersensitive chemiluminescence kit (Feienbio, ES-0006, Wuhan, China) (31).

### Nile red staining

Lipid content was evaluated via Nile Red staining. Briefly, cells were treated for 15 min with Nile Red (1 μM, HY-D0718, MCE) in the dark at 37°C (32, 33). After rinsing with PBS, these cells were counterstained with DAPI (1:2000 in PBS) for 10 min to facilitate nuclear counterstaining (34). After rinsing with HBSS/Ca/Mg, cells were analyzed via fluorescence microscopy (IX73, Olympus or Imager D2, Carl Zeiss). Plasma triglyceride (TG) and total cholesterol (TC) levels were analyzed with Labassay™ kits (Wako, Saitama, Japan) in accordance with provided instructions (35).

### CCK-8 assay

A Cell Counting Kit-8 (CCK-8) assay was performed based on provided instructions to assess cellular viability. Briefly, cells were added to 96-well plates (2,000/well). At 0, 1, 2, 3, and 4 days post-seeding, 10 μL of CCK-8 solution (KeyGEN, Nanjing) was added per well, and absorbance at 450 nm was assessed with a microplate reader (Spark 10M, Shenyang, China). Samples were analyzed in triplicate (36).

### Cell proliferation assay

Cells in appropriate experimental groups were cultured in a monolayer, detached using trypsin, and added to 6-well plates (400-1000 cells/well) followed by incubation for 14 days or until colonies were visible. Media was exchanged every 2-3 days. After colonies had formed, cells were rinsed with PBS and fixed for 15 min with formaldehyde, followed by Giemsa staining for 10-30 min. Running water was then used to rinse plates, followed by sample air-dying. Colonies containing > 50 cells were then counted by eye or under low-level magnification, with colony-forming efficiency being computed as follows: number of colonies/number of seeded cells * 100% (37).

### EdU assay

Cell-Light EdU Apollo®567 *In Vitro* Imaging Kits (RiboBio) were employed to evaluate cellular EdU uptake. Briefly, 2,000 cells were added per well of a 96-well plate and treated with 100 μL of media supplemented with 20 μM EdU. Following incubation for 2 h, cells were fixed for 30 min using 4% paraformaldehyde, rinsed with PBS, and permeabilized using 0.5% Triton X-100 followed by nuclear counterstaining for 30 min using Hoechest 33342 at room temperature. Proliferation rates were then calculated based on provided instructions (Ribo Biotech, Guangzhou), and positively stained cells were assessed with a fluorescence microscope (Leica, Germany) following a PBS wash (38).

### Wound healing assay

A straight line was drawn on the bottom of individual wells of 6-well plates into which cells were then plated and allowed to attach overnight. A 100 uL tip was then used to generate a scratch wound on this line, and PBS was used to rinse the wounded cell monolayer 2-3 times followed by the addition of fresh serum-free media. Cells were then imaged via microscopy after a 24 h incubation, and ImageJ was used to compute the frequency of migrated cells (39).

### Transwell assay

For cell invasion assays, Transwell plates with an 8 μm pore polycarbonate membrane (Corning, USA) were utilized. The upper chambers were seeded with 5 × 10^5^ cells in serum-free media after the chamber had been Matrigel-coated (BD Biocoat, USA). Then, 600 μl of complete media was placed in the lower chamber. Following a 24 h incubation period, cells on the lower side of the membrane were fixed and stained using crystal violet (40).

### Animal studies

The Experimental Animal Care Commission of Sun Yat-sen University approved all animal studies, with were consistent with both the NC3Rs ARRIVE protocols and with institutional ethical guidelines. Tumor xenograft models were established by randomly assigning 4-week-old male nude BALB/c mice from the Sun Yat-sen University Experimental Animal Center to groups, after which they were subcutaneously implanted on the dorsal side of the thigh with 5 × 10^6^ RCC cells. Tumors were measured weekly, and tumor volume (mm^3^) was measured as Volume = 0.5 × length × width^2^. After five weeks, mice were euthanized with CO_2_ and tumors were resected and weighed (41).

### Statistical analyses

R v 4.1.2 was used for statistical analyses and figure construction was conducted with GraphPad Prism v 8.0.1. Kaplan-Meier curves were used to evaluate survival outcomes, and risk model validation was performed with the R ‘survivalROC’ package that enabled the generation of time-dependent ROC curves. Age, sex, clinical stage, and grade were used to conduct subgroup analyses. Continuous data were reported as means ± standard deviation while categorical data were reported as numbers and percentages. Data were compared with t-tests and one-way ANOVAs as appropriate, and P < 0.05 was selected as the cut-off to define significant differences.

## Results

### Identification of lipid metabolism- and OS-related DEGs in ccRCC

**Figure 1:**
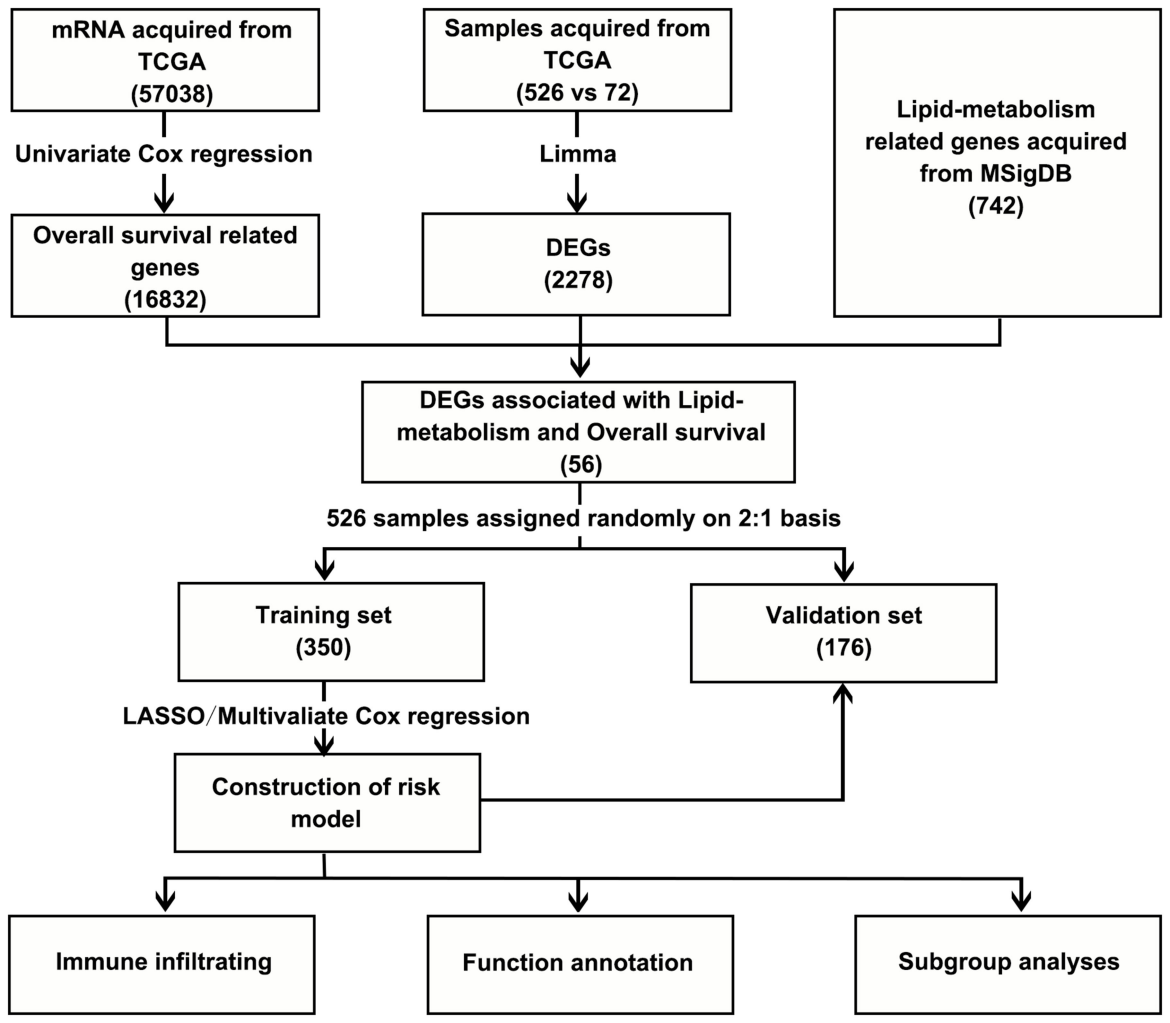
Flow chart.

**Figure 2:**
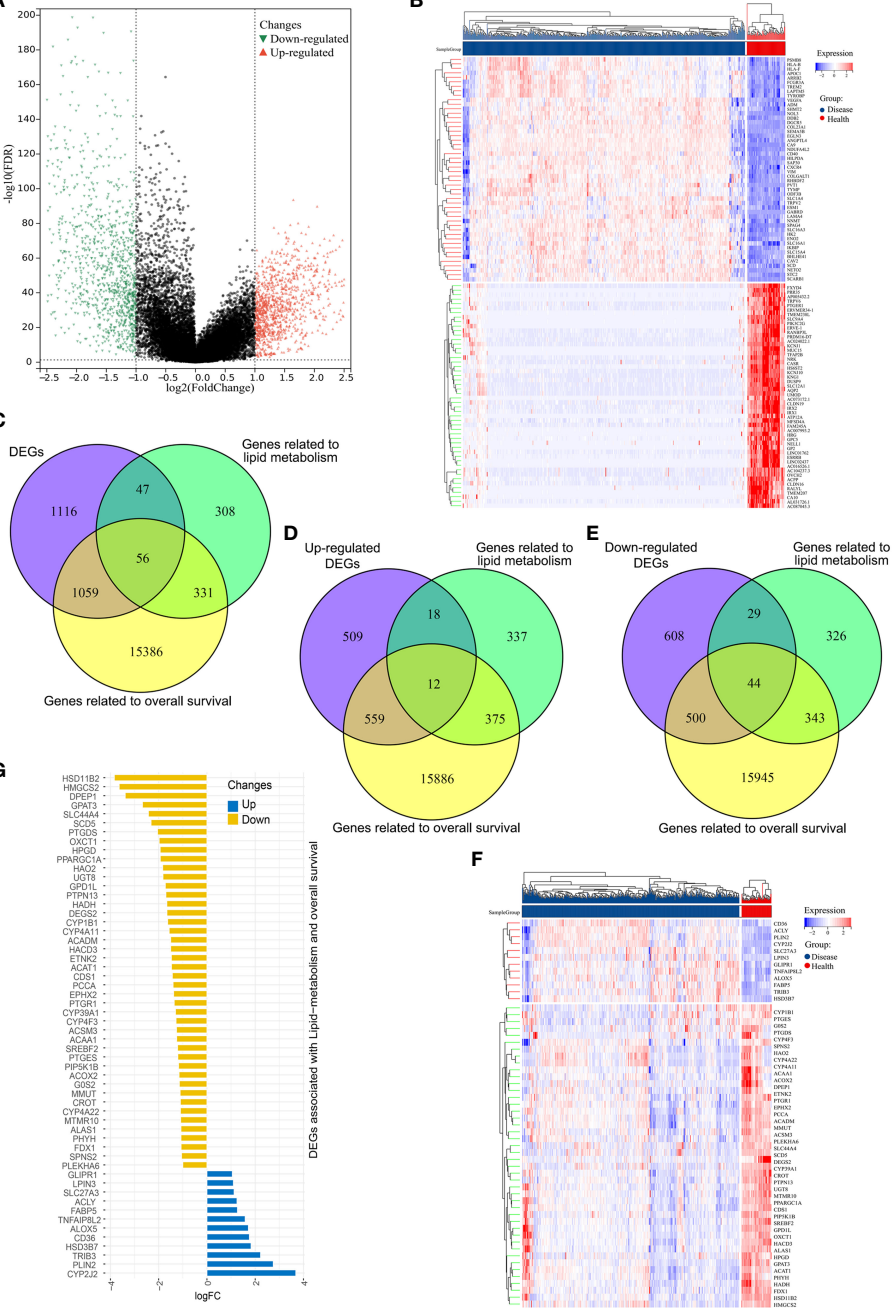
Identification of differentially expressed genes (DEGs) associated with overall survival and lipid metabolism. **(A)** DEGs in ccRCC samples were represented using a volcano plot, with log2(FC) and -log10(adj.P-value) on the x- and y-axes, respectively. Red, green, and black points correspond to genes that were significantly upregulated, significantly downregulated, and not differentially expressed, respectively. **(B)** ccRCC-related DEG expression levels were represented with a heatmap, with dark blue denoting diseased samples, red indicating healthy samples, blue indicating lower levels of gene expression, and red representing higher levels of gene expression. **(C)** A Venn diagram for genes overlapping from three datasets. **(D, E)** Venn diagrams for upregulated **(D)** and downregulated **(E)** DEGs, where purple represents the upregulated/downregulated genes, green denotes lipid metabolism-related genes, and tallow indicates OS-related genes. **(F)** A heatmap of the 56 OS and lipid metabolism-related DEGs in ccRCC, with dark blue denoting diseased samples, red indicating healthy samples, blue indicating lower levels of gene expression, and red representing higher levels of gene expression. **(G)** A deviation map for these 56 DEGs and associated gene expression levels, with upregulated and downregulated DEGs respectively represented in blue and yellow.

Initially, mRNA expression data from 526 ccRCC samples and 72 normal control tissues from the TCGA data for which clinical data were available were downloaded and analyzed (**Figure 1**). These analyses revealed 2278 total DEGs, of which 1098 and 1180 were respectively up- and down-regulated (**Figures 2A, B**). When this DEG list was compared with a list of lipid metabolism- and OS-related genes, 56 total overlapping DEGs were identified of which 44 were downregulated while 12 were upregulated (**Figures 2D, E**). These DEGs were used to generate heat maps and plots highlighting deviations in gene expression between tumors and normal tissues (**Figures 2F, G**).

### Risk model establishment

**Figure 3:**
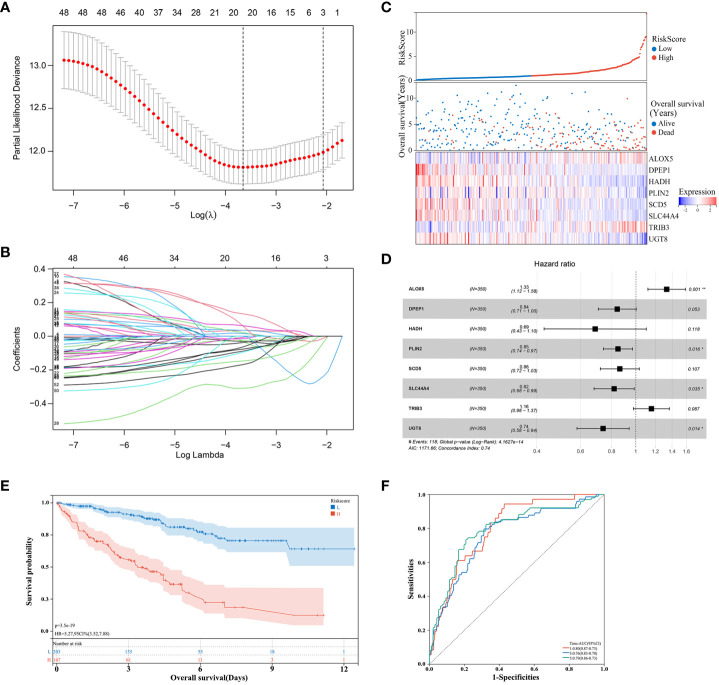
Risk model development in the training cohort. **(A, B)** A LASSO analysis was performed with the minimum lambda value. **(C)** Risk score, survival status, and expression level distributions for 8 risk-related genes in low- and high-risk ccRCC patients. **(D)** Forest plots and corresponding hazard ratios. **(E)** Survival curves for ccRCC patients in both groups. **(F)** Risk model-related time-dependent ROC curve.

Next, the 526 ccRCC tissue samples derived from the TCGA cohort were separated at a 2:1 ratio into training and validation cohorts containing 350 and 176 samples, respectively. A risk signature model was then developed for use in the assessment of ccRCC patient prognostic outcomes based on their expression of OS- and lipid metabolism-related DEGs. A LASSO approach was used to develop the risk model, ultimately identifying 21 genes for incorporation into this model based on an optimal lambda value (**Figures 3A, B**). Based on multivariate Cox analyses of these genes and associated survival analyses, 8 total genes were identified and used for risk model construction including ALOX5, DPEP1, HADH, PLIN2, SCD5, SLC44A4, TRIB3, and UGT8 (**Table 1**). The formula for the final risk model was: Risk Score = ALOX5_expression_ × (0.284602291782237) + DPEP1_expression_ × (-0.173223599727972) + HADH_expression_ × (-0.377221547511465) + PLIN2_expression_ × (-0.165758076212933) + SCD5_expression_ × (-0.148260369122603) + SLC44A4_expression_ × (-0.200778865464863) + TRIB3_expression_ × (0.145106539947866) + UGT8_expression_ × (-0.302444887507542). This model was then employed to separate ccRCC patients into low- and high-risk groups using an optimized cut-off value (**Figure 3C**). High-risk patients exhibited higher ALOX5 and TRIB3 expression levels together with lower DPEP1, HADH, PLIN2, SCD5, SLC44A4, and UGT8 levels as compared to low-risk patients. Consistent with the group names, low-risk patients exhibited superior survival outcomes as compared to high-risk individuals (**Figure 3C**). Forest plots also demonstrated that ALOX5 and TRIB3 were associated with a poor prognosis (HR > 1), whereas the other six genes were associated with a positive prognosis (HR < 1) (**Figure 3D**).

**Table 1:** The eight characteristic genes to construction risk models.

Characteristic genes	Coefficient	HR	HR 95%CI (lower)	HR 95%CI (upper)	P-value
ALOX5	0.284602292	1.329233276	1.116996907	1.581795877	0.001343276
DPEP1	-0.1732236	0.840949558	0.705414201	1.002526115	0.053380774
HADH	-0.377221548	0.685764128	0.42707188	1.10115524	0.118483853
PLIN2	-0.165758076	0.84725118	0.740821628	0.968970848	0.01551239
SCD5	-0.148260369	0.862206594	0.719806361	1.032778051	0.107446757
SLC44A4	-0.200778865	0.81809332	0.679147444	0.98546595	0.03450459
TRIB3	0.14510654	1.156162741	0.979254628	1.365030346	0.086795128
UGT8	-0.302444888	0.739009216	0.580229576	0.941238853	0.014259554

HR, hazard ratio; CI, confidence interval.

Low-risk patients consistently exhibited better OS than high-risk patients (**Figure 3E**). Model diagnostic performance was assessed with ROC curves, revealing acceptable predictive utility. Time-dependent ROC curve analyses of this model revealed an AUC of 0.80, 0.76, and 0.79 at 1, 3, and 5 years, respectively (**Figure 3F**).

**Figure 4:**
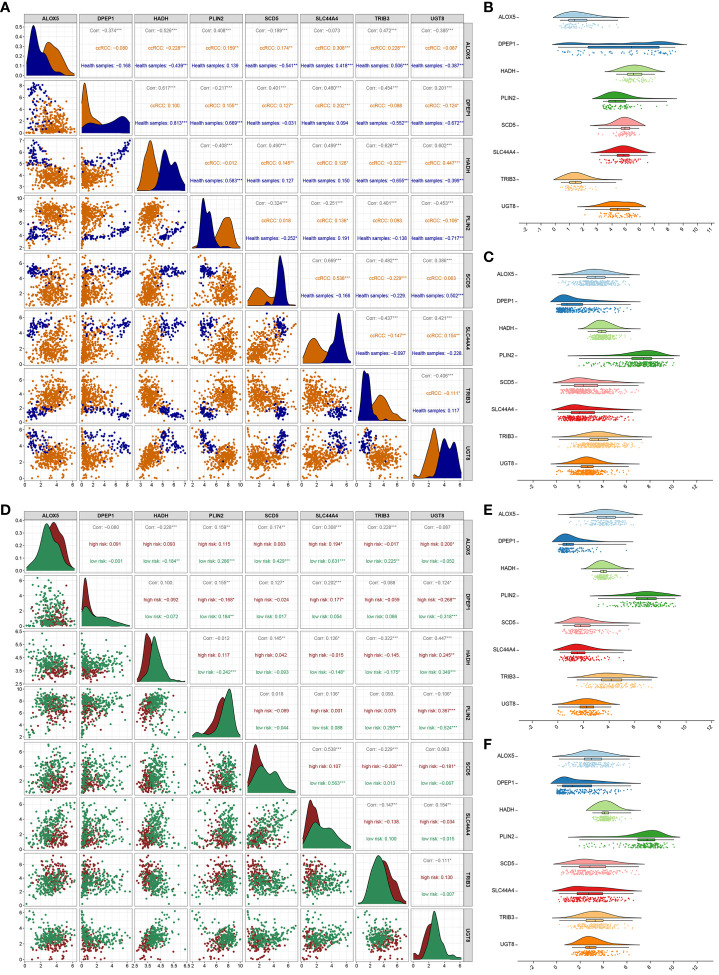
Correlation analysis of 8 genes. **(A)** Analyses of correlations between the expression of the 8 risk genes in healthy control and ccRCC samples. Asterisks represent significance levels for correlations, while numbers correspond to the correlation coefficient. **(B, C)** The distributions of gene expression for the 8 risk genes in healthy **(B)** and ccRCC **(C)** samples. **(D)** Correlation analyses for the 8 risk genes in the low- and high-risk groups. **(E, F)** Distributions of the 8 risk genes in the high-risk **(E)** and low-risk **(F)** groups. *P < 0.05, **P < 0.01, ***P < 0.001.

Next, correlations among the expression of these 8 signature genes were evaluated. In healthy samples, a strong correlation of 0.813 was noted between DPEP1 and HADH, while the correlation between SLC44A4 and SCD5 in ccRCC samples was 0.538 (**Figure 4A**). SCD5 distributions in healthy samples were fairly concentrated, whereas a broader range of DPEP1 expression levels was evident (**Figure 4B**). In ccRCC samples, HADH expression levels were fairly concentrated whereas PLIN2 expression was more variable (**Figure 4C**). The correlation coefficient between PLIN2 and UGT8 was 0.367 in the high-risk group while that between SLC44A4 and ALOX5 in the low-risk group was 0.631 (**Figure 4D**). HADH expression was relatively concentrated while that of PLIN2 was more variable in both the high- and low-risk groups (**Figures 4E, F**).

### Evaluation of the immune status of low- and high-risk ccRCC patients

**Figure 5:**
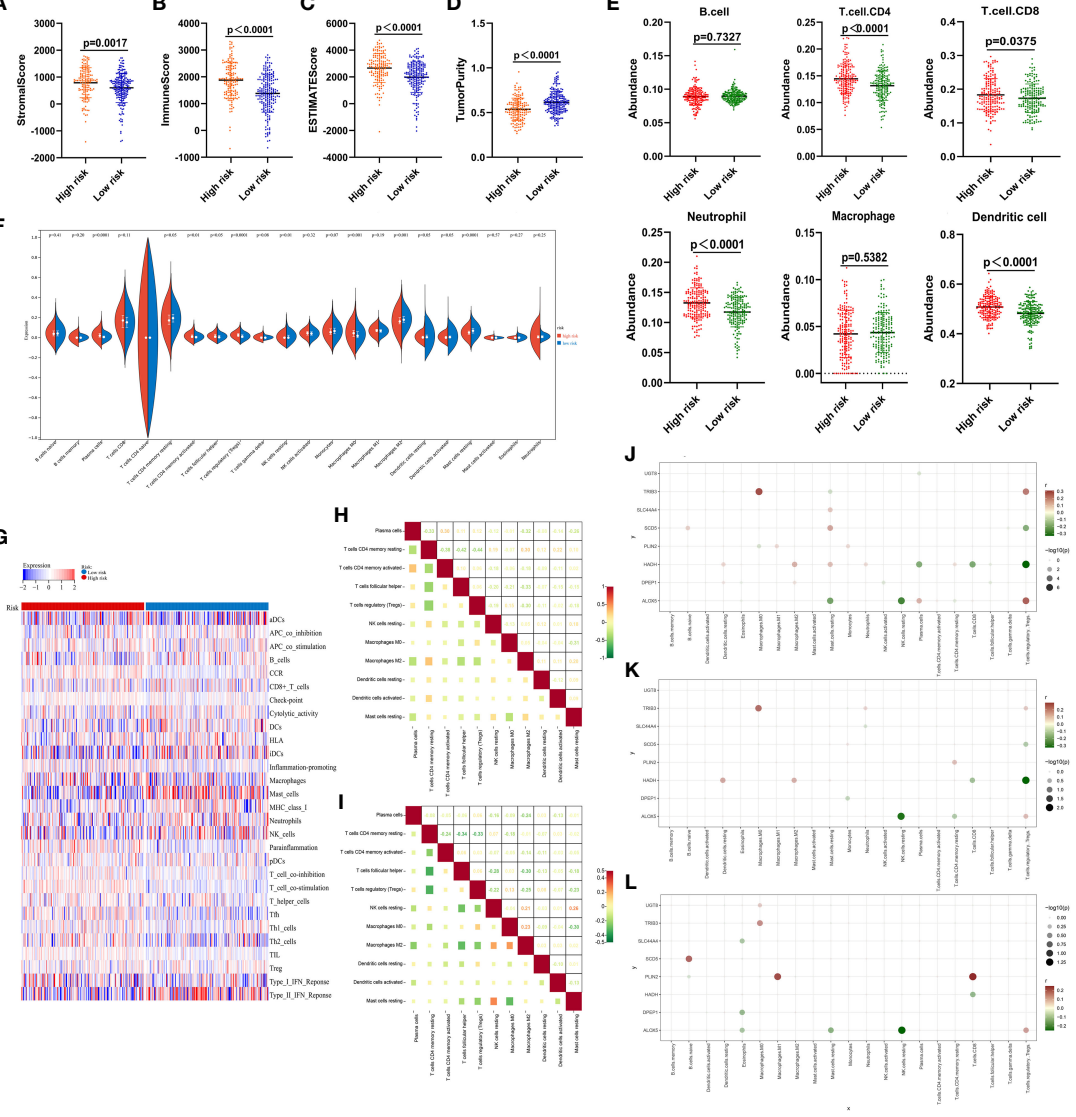
Immune score analyses for low- and high-risk ccRCC patients. **(A–D)** Stromal, immune, and ESTIMATE scores and tumor purity as assessed with the ESTIMATE algorithm. **(E)** The degree of infiltration by 6 immune cell types as estimated with the TIMER algorithm. **(F)** Comparisons of infiltration by 22 different immune cell types between low- and high-risk ccRCC samples. **(G)** Enrichment levels for 29 immune-related gene sets are shown in a heat map as determined with the ssGSEA algorithm. **(H)** Correlations were analyzed for the 11 immune cell populations that differed significantly between groups in **(F)**, with numbers representing correlation coefficients. **(J–L)** Correlations between the 8 risk-related genes and immune cell populations in the overall ccRCC patient cohort **(J)**, high-risk patients **(K)**, and low-risk patients **(L)**. All 21 immune cell types other than T.cells.CD4.naive are shown on the horizontal axis, and the vertical axis represents the 8 risk-related genes. The presence of a node indicates a significant correlation (P < 0.05). Node color is indicative of correlation strength, while node size is proportional to significance level.

These data support the value of the developed risk model as a tool for the prognostic evaluation of patients with ccRCC. To assess the relationship between this risk model and patient immune status, the ESTIMATE algorithm was used, revealing that high-risk patients exhibited a significantly higher stromal score (P=0.0017), immune score (P<0.0001), and ESTIMATE score (P<0.0001), together with lower levels of tumor purity (P<0.0001) relative to the low-risk group (**Figures 5A–D**. The TIMER algorithm also revealed significantly increased in CD4 T cell (P<0.0001), CD8 T cell (P=0.0375), neutrophil (P<0.0001), and DC (P<0.0001) levels in high-risk samples, whereas no differences in B cell (P=0.7327) or macrophage (P=0.5382) levels were evident (**Figure 5E**). An analysis of 22 different infiltrating immune cell types in these risk groups revealed significant differences for 11 of these cell types, including resting memory CD4 T cells, activated memory CD4 T cells, T follicular helper (Tfh) cells, regulatory T cells (Tregs), resting NK cells, M0 macrophages, M2 macrophages, resting DCs, activated DCs, and resting mast cells (**Figure 5F**).

Clear differences in the immune landscape, as identified through a ssGSEA analysis (**Figure 5G**), were evident when comparing these risk groups. Correlation analyses of the 11 significant immune cell types revealed that CD4 T cells and Tregs were negatively correlated in the high-risk group (r = -0.44) (**Figure 5F**), while M0 macrophages and resting mast cells were respectively negatively and positively correlated with most immune cell types (**Figure 5H**). Resting memory CD4 T cells were also correlated with Tfh cells in the low-risk group (r = -0.34). Plasma cells, as well as resting and activated memory CD4 T cells, were negatively correlated with most cell populations (**Figure 5I**).

Strong correlations were also noted between the 8 risk-related genes and different immune cell populations in the overall ccRCC patient cohort (**Figure 5J**), high-risk patients (**Figure 5K**), and low-risk patients (**Figure 5L**). None of these genes were correlated with B cells, DCs, activated mast cells, or gamma delta T cells (**Figure 5J**), while resting cells were only somewhat correlated with these genes. Similar results were evident in both the low- and high-risk groups (**Figures 5K, L**). The observed correlations suggest that lipid metabolism is likely to be associated with immune functionality in ccRCC.

### Functional analyses

**Figure 6:**
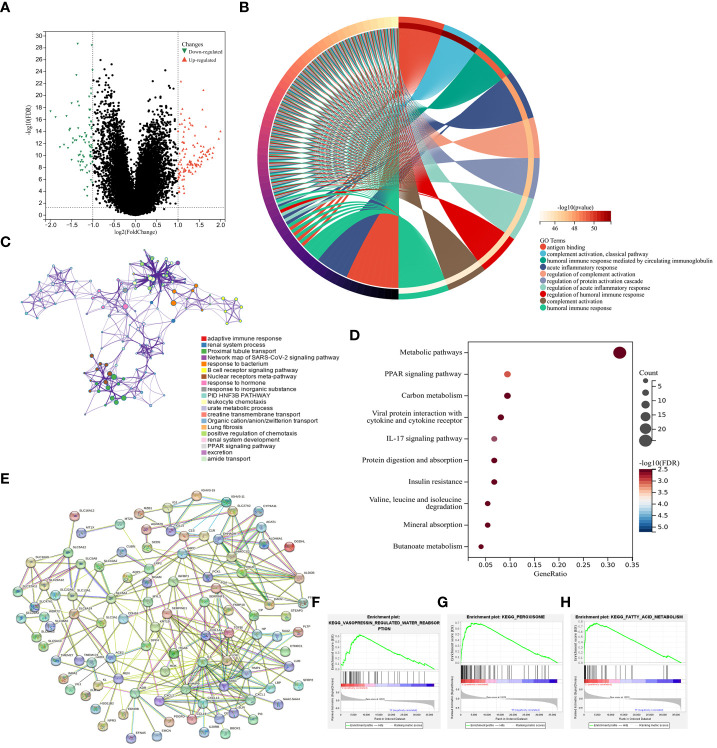
Functional analyses. **(A)** DEGs identified when comparing low- and high-risk groups were presented with a Volcano plot. **(B)** A circle plot was used to visualize enriched GO biological processes associated with these DEGs. **(C)** Enriched GO biological processes were presented in a network visualization. **(D)** Enriched KEGG pathways associated with these DEGs were presented in a bubble diagram. **(E)** DEGs were used to conduct a PPI analysis. **(F–H)** GSEA plots were used to visualize GSEA analysis results.

Next, a new set of 192 DEGs was identified through a comparison of the low- and high-risk groups that included 77 and 115 down- and up-regulated genes, respectively (**Figure 6A**). GO enrichment and network analyses demonstrated that these DEGs were enriched in a range of immune-associated pathways including the antigen binding and complement activation pathways (**Figures 6B, C**). In a similar vein, KEGG enrichment analyses indicated that these genes were enriched in substance metabolism and immune response pathways including the PPAR signaling, metabolism of xenobiotics by cytochrome P450, and fatty acid degradation pathways (**Figure 6D**). In a protein-protein interaction (PPI) analysis (42), IL-6 and MMP9 were closely related to the immune response, supporting key roles for lipid metabolism and immune activity in ccRCC development (**Figure 6E**). In line with these data, GSEA analyses revealed that the most strongly enriched stable internal environment, peroxisome, and fatty acid metabolism pathways were expressed at low levels in the high-risk group (**Figures 6F–H**). Together these results suggest that lipid metabolism- and OS-related genes are related to substance metabolism and the immune response in ccRCC, contributing to poor patient outcomes.

### The clinical implications of patient risk scores

**Figure 7:**
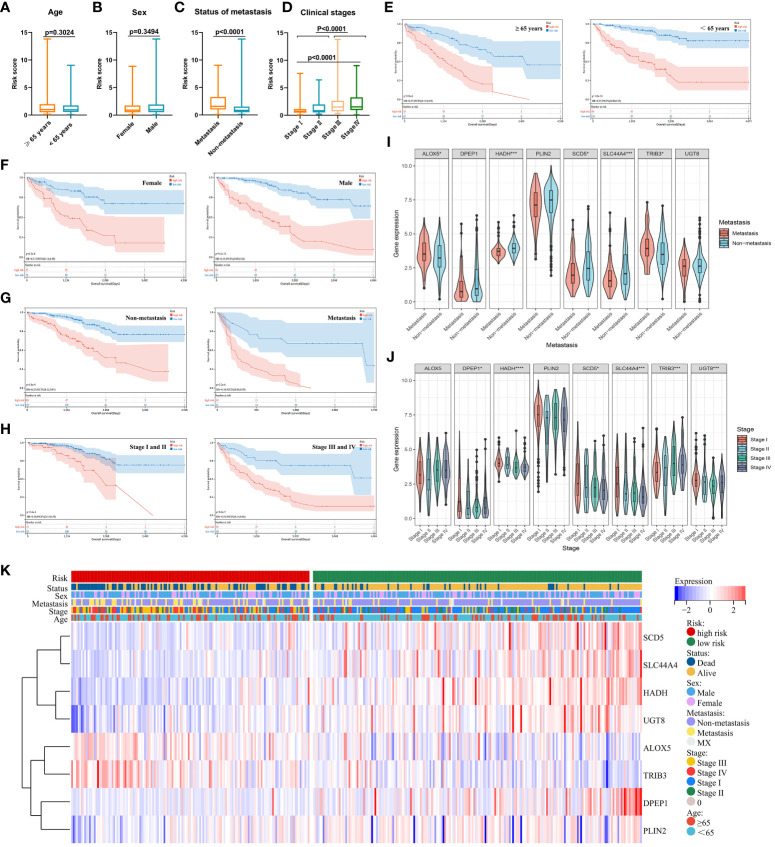
**(A–D)** Relationships between risk scores and clinical characteristics were evaluated, revealing that no differences in ccRCC patients were evident as a function of age **(A)** or sex **(B)**, while significant differences were evident with respect to metastatic status **(C)** and clinical staging **(D)**. **(E–H)** Risk model independence was evaluated by initially generating survival curves for patients grouped according to age **(E)**, sex **(F)**, metastatic status **(G)**, and clinical stage **(H)**. **(I–K)** Associations between genes and clinical characteristics including metastatic stage **(I)**, clinical stage **(J)**, and multiple clinical variables **(K)** were assessed. *P < 0.05, ***P < 0.001, ****P < 0.0001.

Next, risk score value associations with patient clinical characteristics were assessed in the training, validation, and prediction cohorts (**Table 2**). In training set, no differences in age or sex were detected as a function of risk score (**Figures 7A, B**), whereas both metastatic status and clinical stage significantly differed based on risk score (**Figures 7C, D**). When these ccRCC patients were grouped based on age (**Figure 7E**), sex (**Figure 7F**), metastatic status (**Figure 7G**), and clinical stage (**Figure 7H**), the predictive performance of the risk model remained intact such that low-risk patients exhibited better outcomes. In Cox regression analyses, this risk model independently predicted ccRCC patient prognosis (**Figures 7E–H**). Together, these data demonstrate that this risk model can effectively assess ccRCC patient outcomes independent of other clinical characteristics.

**Table 2:** Characteristics of patients in the training, validation, and prediction set.

		Total (N=526)	Training set (N=350)	Validation set (N=176)	Prediction set (N=101)	P_TV_-value	F_TV_-value
		n/%	n/%	n/%	n/%		
Age						0.0818	0.0855
	<65years	332/65.97	230/65.71	102/57.95	52/51.49		
	≥65years	194/34.03	120/34.29	74/42.05	49/48.51		
Sex						0.1606	0.1750
	Female	183/34.79	129/36.86	54/30.68	23/22.77		
	Male	343/65.21	221/63.14	122/69.32	78/77.23		
Survival Status						0.3347	0.3742
	Alive	356/67.6	232/66.29	124/70.45	78/77.23		
	Dead	170/32.3	118/33.71	52/29.55	23/22.77		
Clinical Stages	StageI	261/49.62	171/48.86	90/51.14	67/66.34		
	StageII	57/10.84	31/8.86	26/14.77	11/10.89		
	StageIII	123/23.38	80/22.86	43/24.43	22/21.78		
	StageIV	82/15.59	65/18.57	17/9.66	1/0.99		
	Not Available	3/0.57	3/0.86	0/0	0/0		
						0.0885	0.1070
	StageI~II	318/60.46	202/57.71	116/65.91	78/77.23		
	StageIII~IV	205/38.97	145/41.43	60/34.09	23/22.77		
Pathological Grading	Grade1	13/2.47	8/2.29	5/2.84	13/12.87		
	Grade2	226/42.97	153/43.71	73/41.48	58/57.43		
	Grade3	205/38.97	129/36.86	76/43.18	23/22.77		
	Grade4	74/14.07	53/15.14	21/11.93	5/4.95		
	Not Available	8/1.52	7/2.00	1/0.57	2/1.98		
						0.6092	0.6418
	Grade1~2	239/45.44	161/46.00	78/44.32	71/70.30		
	Grade3~4	279/53.04	182/52.00	97/55.11	28/27.72		

The P_TV_-value and F_TV_-value were obtained, respectively, from the age, sex, Survival Status, Clinical Stages (StageI~II and StageIII~IV), and Pathological Grading (Grade1~2 and Grade3~4) between the training and validation set.

When assessing the association between expression levels for particular genes and patient clinical characteristics, significant differences in ALOX5, HADH, SCD5, SLC44A4, and TRIB3 expression were detected when comparing patients with metastatic and non-metastatic disease (**Figure 7I**) In addition, differences in DPEP1, HADH, SCD5, SLC44A4, TRIB3, and UGT8 levels were noted among patients with different clinical stages of disease (**Figure 7J**). Associations between genes and clinical characteristics were presented with a heat map (**Figure 7K**), revealing the overexpression of ALOX5 and TRIB3 in high-risk ccRCC patients such that they may play important oncogenic roles. High-risk patients also presented with higher rates of mortality, later clinical staging, and a higher likelihood of having metastatic disease. The other 6 genes in the risk model were expressed at lower levels in high-risk ccRCC patients.

### Evaluation of ccRCC risk model diagnostic efficacy and prognostic performance

**Figure 8:**
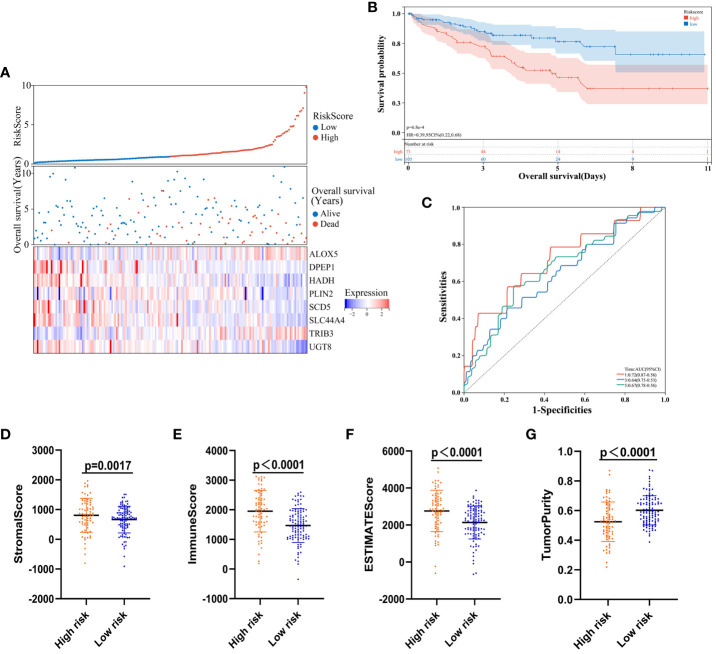
Risk model validation. **(A)** Risk score, survival status, and gene expression level distributions for low- and high-risk ccRCC patients in the validation cohort. **(B)** Survival curves demonstrating low- and high-risk patient outcomes for the validation cohort. **(C)** Model time-dependent ROC curves for the validation cohort. **(D-G)**Stromal, immune score, ESTIMATE, and tumor purity calculations for the validation cohort.

Next, developed risk score model performance was assessed in the validation cohort. Patients were stratified into high- and low-risk groups using the formula established above, and the expression of the eight risk score candidate genes was presented in these groups with a heat map (**Figure 8A**). Survival analyses confirmed that high-risk patients in this cohort faced worse prognostic outcomes (**Figure 8B**), and time-dependent ROC curves revealed that the AUC values for this model when predicting 1-, 3-, and 5-year survival were 0.72, 0.64, and 0.67, respectively (**Figure 8C**). Individuals in the high-risk group also exhibited a significantly higher stromal score (P = 0.0017), immune score (P < 0.0001), and ESTIMATE score (P < 0.0001) relative to low-risk individuals (**Figures 8D–F**), whereas their tumor purity was significant below that for low-risk patients (P < 0.0001; **Figure 8G**).

To assess the universality of the developed risk model beyond the training cohort, we introduced an independent cohort from E-MTAB-1980 as the prediction group. Survival analyses validated that individuals classified as high-risk in this cohort experienced poorer prognostic outcomes (**Figure S1A**). Additionally, time-dependent ROC curves illustrated the model’s predictive performance for 1-, 3-, and 5-year survival, with corresponding AUC values of 0.82, 0.76, and 0.75, respectively (**Figure S1B**).

These findings confirmed the excellent diagnostic efficacy and performance of the established model, given its close association with ccRCC patient outcomes and immune status in the validation and prediction cohort.

### Nomogram development and calibration

**Figure 9:**
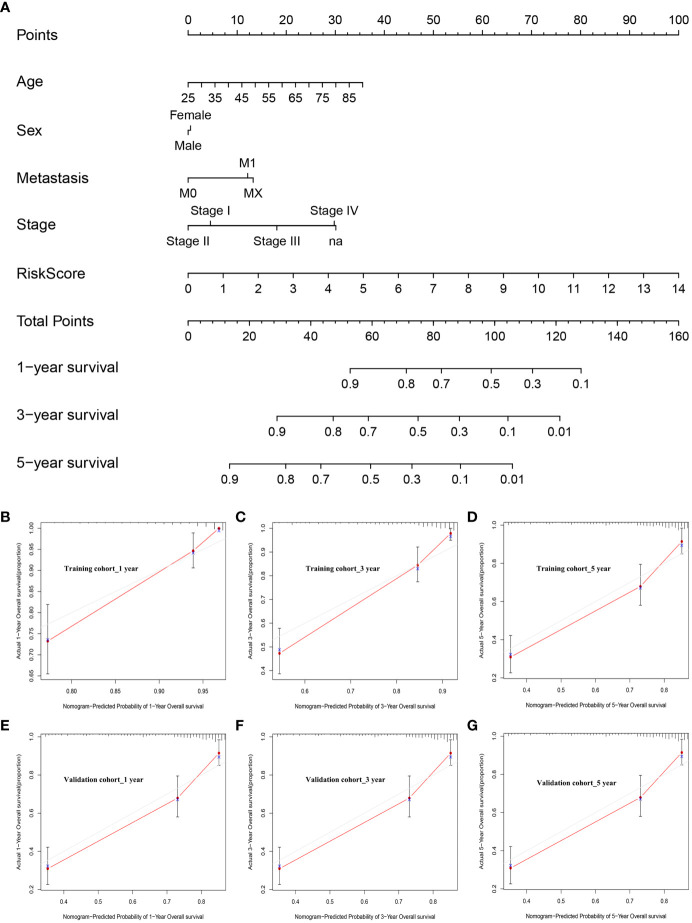
Nomogram development and calibration. **(A)** The established nomogram incorporating risk score values and clinical characteristics. **(B–D)** Nomogram calibration in the training cohort at 1, 3, and 5 years. **(E–G)** Nomogram calibration in the validation cohort at 1, 3, and 5 years.

To build on the promising data presented above, patient risk scores were integrated with clinical characteristics and used to develop a nomogram capable of more reliably predicting prognostic outcomes (**Figure 9A**). In this nomogram, scores were assigned in accordance with patient risk scores and other clinical characteristics, and the total score was used to gauge their odds of survival. Calibration curves and decision curve analyses for this nomogram in both cohorts suggested that its accuracy was acceptable (**Figures 9B–G**). Specifically, the predicted and actual OS for these patients aligned well at 1, 3, and 5 years in the training (**Figures 9B–D**) and validation (**Figures 9E–G**) cohorts. As such, this nomogram was capable of effectively predicting ccRCC patient outcomes. Ultimately these data support the important role that dysregulated lipid metabolism plays in shaping poor ccRCC patient outcomes such that a risk model based on dysregulated OS- and lipid metabolism-related genes was capable of reliably predicting the prognosis of these patients.

### Validation of risk-related gene expression in ccRCC

**Figure 10:**
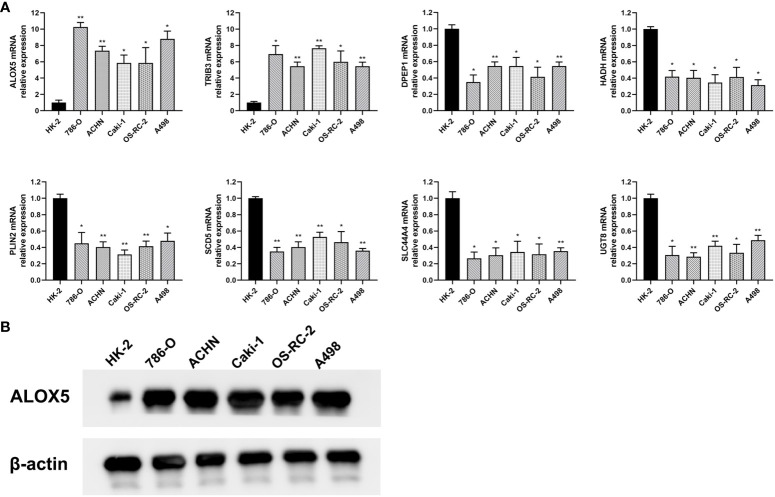
Validation of differential risk-related gene expression. **(A)** The mRNA level expression of ALOX5, DPEP1, HADH, PLIN2, SCD5, SLC44A4, TRIB3, and UGT8 was assessed via qPCR in the 786-O, ACHN, Caki-1, OS-RC-2, and A498 ccRCC cell lines and in control HK-2 cells. **(B)** ALOX5 protein levels in ccRCC cells were detected via Western immunoblotting. Data are means ± SD. *, P<0.05; **, P<0.01.

To confirm the bioinformatics predictions discussed above, the expression levels of the 8 risk-related genes were assessed in ccRCC samples via qPCR and Western immunoblotting. Primers used for these qPCR analyses are presented in **Table 3**. At the mRNA level, significantly increased *ALOX5* and *TRIB3* expression was evident in ccRCC samples, whereas the opposite was evident for *DPEP1, HADH, PLIN2, SCD5, SLC44A4*, and *UGT8*, with differential expression being most significant for *ALOX5* (**Figure 10A**). These data were consistent with the reliability of the bioinformatics results on which the risk model was based. Western blotting was also used to detect ALOX5 protein levels in the 786-O, ACHN, Caki-1, OS-RC-2, and A498 human ccRCC cell lines and the control HK-2 cell line, ultimately revealing that this protein was upregulated in ccRCC cells relative to HK-2 cells (**Figure 10B**), consistent with results observed at the mRNA level. Moreover, we conducted PCR and immunohistochemistry (IHC) staining on 12 clinical ccRCC tissue specimens. The resultes revealed an overexpression of ALOX5 mRNA levels in human ccRCC specimens (**Figure S2A**), consistent with the observed results in cell lines. Utilizing IHC, we assessed ALOX5 protein expression levels in ccRCC tumor tissues and normal tissues. As illustrated in **Figure S2B**, our study demonstrated intense ALOX5 immunostaining signals in the ccRCC cells, while such signals were weaker in healthy renal tissues. The IHC score of ccRCC tumor tissues exceeds that of normal tissues, signifying statistically significant distinctions between these two tissue groups. (**Figure S2C**).

**Table 3:** PCR primers.

Characteristic genes	Forward primer sequence	Reverse primer sequence
ALOX5	TGGCGCGGTGGATTCATAC	CCAGTCGTCATTCAGCCAGT
DPEP1	GACAGCCTGGTGATGGTGAA	TGTTCCACAGCCTCGAAGAC
HADH	AGCTAATGCCACCACCAGAC	AGCCCAAACCCGAGTTAGAA
PLIN2	TGATGGCAGGCGACATCTAC	CTGGCTGCTCTTGTCCATCT
SCD5	GGTGCTCATGTGCTTTGTGG	GTCATAGGGCCGGTTTCCAT
SLC44A4	TTCGAGGCCCCATCAAGAAC	CTTGTGATCACCGTCTGGGG
TRIB3	TGCGTGATCTCAAGCTGTGT	GCTTGTCCCACAGGGAATCA
UGT8	GGATCAACCTGGTCACCCTG	GGAGATCTGATGGACAGCGG
β-actin	AGGATTCCTATGTGGGCGAC	ATAGCACAGCCTGGATAGCAA

### Functional validation of risk-related genes in ccRCC

**Figure 11:**
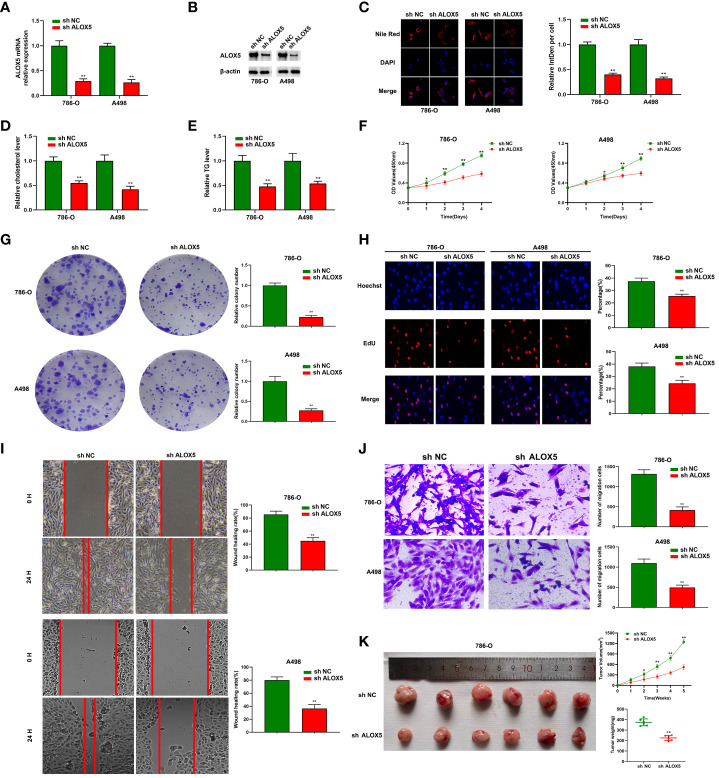
ALOX5 promotes abnormal lipid metabolic activity, proliferation, migratory activity, and invasivity in ccRCC cells. **(A, B)** ALOX5 knockdown was associated with a reduction in the expression of this gene in 786-O and A498 cells at the mRNA **(A)** and protein **(B)** levels. **(C)** Nile Red staining was performed to detect lipids in ccRCC cells and the results were quantified. **(D, E)** Total cholesterol and triglyceride levels were measured following ALOX5 knockdown. **(F–J)** Knocking down ALOX5 suppressed ccRCC cell proliferation **(F)**, colony formation **(G)**, EdU uptake **(H)**, wound healing activity **(I)**, and Transwell invasion **(J)** *in vitro.* **(K)** Knocking down ALOX5 suppressed *in vivo* ccRCC tumor growth, with tumor volume having been measured every third day for 5 weeks post-implantation. *P<0.05; **P<0.01.

To confirm the functional importance of ALOX5 in ccRCC, it was next knocked down, with knockdown efficiency in 786-O and A498 cells being successfully confirmed through qPCR and Western immunoblotting (**Figures 11A, B**). Total cholesterol and triglyceride accumulation in these cells was then assessed through Nile Red staining and confocal imaging (**Figure 11C**). In these analyses, lipids were red while nuclei were blue. Total cholesterol and triglyceride levels in these cells were also quantified (**Figures 11D, E**). To further assess the functional importance of ALOX5 in these two cell lines, a series of functional analyses were performed. The knockdown of ALOX5 impaired cell proliferation (**Figure 11F**) and colony formation (**Figure 11G**). Similarly, EdU uptake was impaired with ALOX5 silencing (**Figure 11H**), indicating a reduction in DNA synthesis activity. Knocking down ALOX5 also impaired the migratory and invasive activity of these ccRCC cells in wound healing and Transwell assays (**Figures 11I, J**). In line with these *in vitro* data, reduced ALOX5 expression was associated with the significant impairment of xenograft tumor growth in tumor-bearing mice (**Figure 11K**).

## Discussion

Here, DEGs associated with lipid metabolism and ccRCC patient OS were used to establish a prognostic risk model capable of predicting outcomes for these patients with a high degree of accuracy. Model-derived risk scores were employed to stratify patients into low- and high-risk groups. Patients in these two groups exhibited distinct immune landscapes and significantly different survival outcomes. Those patients exhibiting a poorer prognosis tended to present with higher immune and ESTIMATE scores as well as reduced tumor purity relative to patients that experienced better outcomes. Functional analyses demonstrated that reduced immune activity was associated with a stable internal environment, peroxisomes, and fatty acid metabolism. ALOX5 was also selected as a functional gene associated with these phenotypic results for subsequent validation. Together, these results will aid in the design of new treatments for ccRCC such that clinicians can make more informed and effective treatment-related decisions.

In an effort to clarify the molecular factors that govern the onset and progression of ccRCC, interactions between tumor gene expression and the tumor-associated microenvironment were also explored (2, 43). The ESTIMATE algorithm enables the approximation of tumor purity and levels of immune cell infiltration based upon gene expression data (44). In one prior report, Xu et al. noted that lower tumor purity and higher immune score values were associated with poorer ccRCC patient prognostic outcomes (15). Consistently, the present data revealed that patients who experienced worse prognostic outcomes had lower tumor purity and higher immune score values. TIMER and ssGSEA approaches were similarly employed to compare the immune status of low- and high-risk patients. Of the six immune cell types analyzed with the TIMER tool (45), four were found to exhibit significantly higher abundance within the tumors of high-risk ccRCC patients, in line with the increases in immunological gene expression noted in ESTIMATE analyses. The ssGSEA approach also enabled the analysis of 28 different immune-associated cell types, ultimately demonstrating that the immune status of high-risk patients was better than that of low-risk patients. Higher immune scores and a more robust immune status thus appear to be correlated with poor prognostic outcomes in ccRCC.

Next, functional analyses were performed with the goal of clarifying the biological mechanisms underlying the results evident for these two risk groups. Identified DEG-based GO, KEGG, and PPI analyses revealed that dysregulated lipid and fatty acid metabolism may be related to ccRCC tumor development and progression. ssGSEA analyses were thus used to explore these mechanisms in greater detail (46). This approach revealed that high-risk ccRCC patient samples exhibited enrichment for a stable internal environment, peroxisomes, and fatty acid metabolism relative to low-risk patients (19). Dysregulated fatty acid metabolism and lipid catabolism were thus closely tied to higher immune status and worse prognostic outcomes for these patients. Consistently, a growing body of work has focused on the intratumoral dysregulation of lipid metabolism, suggesting that efforts to target lipid-related pathways may enable novel antitumor interventions. Lipid accumulation may also modulate TIME composition in a manner that contributes to worse patient outcomes owing to the storage of lipids in surrounding cells. Malignant tumors and Tregs have widely been shown to promote T cell senescence in various cancers (47). Tumor cells are also equipped to persist in challenging microenvironments such as under conditions of hypoxia or nutrient depletion, or in distant tissues following metastatic dissemination. Complex interactive associations between the TIME, tumor cells, and the stroma ultimately shape lipid use by these transformed cells (48). The present data highlight a mechanistic association between T cell senescence and regulation of lipid metabolism in the TIME, highlighting this avenue as a viable target for immunotherapeutic intervention.

To further confirm the impact of dysregulated lipid metabolism on the TIME in ccRCC and to assess the prognostic relevance of DEGs in affected patients, DEGs associated with survival and lipid metabolism were identified and employed to construct a prognostic risk scoring model. This model incorporated eight genes closely tied to tumor progression. For example, ALOX5 is an enzyme that facilitates the biosynthesis of the arachidonic acid-derived inflammatory mediators known as leukotrienes. Elevated ALOX5 expression is associated with diminished survival in RCC (49). In breast cancer, ALOX5 expression within neutrophils reportedly promotes metastasis to the lungs (50). PLIN2 is a protein associated with adipose differentiation through its ability to regulate lipid storage and metabolism within cells. PLIN2 can facilitate lipid droplet mobilization and thereby regulate a range of processes including the homeostasis of phospholipids, mitochondrial activity, and the lipid remodeling-mediated deacetylation of histones (51). SLC44A4 (solute carrier family 44 member 4) is a membrane transporter that is primarily expressed in the pancreas, liver, and small intestines and that has been linked to lung, prostate, and colorectal cancers (52). Meanwhile, SLC44A4 was found to be significantly associated with OS and disease-free survival (DFS) in ccRCC patients (53). UGT8 (UDP glycosyltransferase 8) encodes an enzyme involved in the phase II metabolic reaction known as glucuronidation. High UGT8 expression has been observed in triple-negative breast cancer to promote greater tumor progression (54). But, UGT8 needs further study inRCC. DPEP1 (dipeptidase 1) codes for a cytoplasmic membrane enzyme responsible for regulating proliferative, metabolic, and differentiation activity. Shi et al. discovered that DPEP1, along with six other genes from the classifier, could be valuable for molecular subtyping and guiding therapy in ccRCC (55). DPEP1 expression has significant increases in B-cell acute lymphoblastic leukemia that are related to progressive and relapsing disease (56). HADH (hydroxyacyl-coenzyme A dehydrogenase) codes for a protein involved in glucose and fat metabolism. HADH dysregulation is also reportedly related to oncogenic processes (57). SCD5 (stearoyl-CoA desaturase 5) encodes a regulator of fatty acid metabolism. The regulation of SCD5 by von Hippel-Lindau impacts the proliferation and lipid homeostasis of ccRCC cells, suggesting a novel mechanism in the formation and progression of ccRCC tumors (58). SCD5 downregulation is evident in advanced melanoma, and restoring its expression can suppress malignancy via decreasing protease and ECM-related protein secretion (59). TRIB3 (tribbles pseudokinase 3) reportedly plays important roles in processes such as growth, differentiation, and metabolism. TRIB3 expression was notably higher in RCC tissues in comparison to paracancerous tissues, and elevated TRIB3 expression correlated with advanced tumor stage and an unfavorable prognosis. Furthermore, TRIB3 knockdown significantly impeded RCC cell proliferation, migration, and invasion (60). In colorectal cancer, TRIB3 plays an inhibitory role while also suppressing CD8 T cell infiltration and promoting glioblastoma cell stem-like properties through interactions with beta-catenin that facilitate tumor growth (61). Here, these 8 genes differed significantly in terms of expression and distribution between ccRCC patients and healthy controls, as well as between high-risk and low-risk groups. Consequently, the resultant risk model was robustly predicted the survival of ccRCC patients and accurately stratified them into high-risk or low-risk categories in both the training and validation cohorts. Subgroup analyses also demonstrated the ability of this model to predict ccRCC patient risk independent of age or gender. The resultant risk scores were combined with clinical characteristics and used to establish a nomogram. Together, these analyses effectively demonstrate the prognostic relevance of these DEGs in ccRCC while emphasizing the close relationship between dysregulated lipid metabolism and TIME composition. These 8 genes may thus be invaluable biomarkers for the diagnosis or prognostic assessment of ccRCC.

The eight risk-related genes identified above were also found to be closely associated with TIME composition in ccRCC patients. These tumor-associated immune cells serve as important regulators of tumorigenic processes. High-risk ccRCC patients exhibited an increase in tumor sample infiltration by plasma cells, activated memory CD4 T cells, Tfh cells, Tregs, and M0 macrophages, whereas low-risk patient samples exhibited infiltration by higher levels of resting memory CD4 T cells, resting NK cells, M2 macrophages, resting DCs, activated DCs, and resting mast cells. This suggests that the cells enriched in high-risk patient samples may promote tumor development, whereas those enriched in low-risk patients may suppress tumorigenesis. These data are consistent with recent research evidence (17, 62). The above results strongly suggest that ALOX5 may be capable of modulating the ccRCC-associated immune microenvironment by influencing immune cell populations capable of promoting tumor growth including plasma cells, activated memory T cells, Tfh cells, Tregs, and M0 macrophages, resulting in the impairment of normal lipid metabolism and poor prognostic outcomes. TRIB3 may similarly contribute to worse outcomes. In contrast, DPEP1, HADH, PLIN2, SCD5, SLC44A4, and UGT8 may function to suppress tumor growth via the regulation of immune cells with antitumor activity such as resting memory CD4 T cells, resting NK cells, M2 macrophages, resting and activated DCs, and resting mast cells. Of these 8 genes, 3 have been reported in prior studies including ALOX5, TRIB3, and PLIN2. Weiger et al. explored possible pathways whereby COX-2/mPGES-1 and ALOX5/-15 expressed in macrophages may contribute to oncogenic progression (63). Moreover, Matareed et al. demonstrated that in uveal melanoma, PLIN2 expression levels were associated with patient survival (64). Further research will be essential to further clarify the link between PEP1, HADH, SCD5, SLC44A4, and UGT8 and the progression or development of ccRCC.

The research (65) showed that ALOX5, one of extracellular vesicle derived mRNA transcripts, was found specific to urine and tumor tissue samples and defined disease-specific extracellular vesicle biomarkers in liquid biopsy patient samples. Also, Wang (66) et al. found higher expression of ALOX5 predicts reduced survival in tumours correlates with worse prognosis in RCC patients. Their integrated analysis illustrated the four hub genes, including ALOX5, involved in RCC tumorigenesis, shedding light on the development of prognostic markers and further understanding of the function of the identifed RCC hub genes could provide deep insights into the molecular mechanisms. These were consistent with our bioinformatics results. Furthermore, we were used to select ALOX5 as a target for subsequent validation in a series of molecular biology assays *in vitro* and *in vivo.* Significant increases in ALOX5 expression were evident in analyzed ccRCC cell lines relative to the control HK-2 cell line corresponding to human proximal tubule epithelial cells. In a loss-of-function assay, silencing ALOX5 in the 786-O and A498 ccRCC cell lines impaired their ability to proliferate, migrate, and engage in invasive activity *in vitro* and *in vivo.*

This analysis has several strengths over prior ccRCC-focused research efforts. For one, this study focused specifically on OS- and lipid metabolism-related DEGs when constructing a ccRCC-specific risk model using LASSO analyses and loop grouping simultaneously, ultimately revealing 8 characteristic risk-related genes significantly associated with TIME composition and prognostic outcomes in these cancer patients. Moreover, differences in biological activity associated with risk scores were comprehensively assessed through a variety of functional enrichment and immune infiltration analyses methods, and the impact of lipid metabolism on TIME composition and patient prognosis was validated. These findings offer an evidence-based foundation for additional research exploring the mechanisms driving ccRCC development and progression, potentially aiding the individualized diagnosis and treatment of this devastating disease. Importantly, these findings were also validated *in vitro* and *in vivo* using ccRCC tumor cells, providing support for these bioinformatics results.

There are certain limitations to this study that warrant further experimental follow-up. For one, more external validation was available for the established risk model owing to the incompleteness of most mRNA and clinical datasets. In addition, only relatively simplistic analyses of ccRCC cells and xenograft tumors were performed herein, underscoring an opportunity for more detailed investigations in the future. While the goal of these analyses was to establish the biological landscape of ccRCC and to identify means of predicting patient risk, the number of samples included herein was still limited. In the future, large-scale and single-cell-based validation will thus be vital. Further multi-omics analytical approaches such as lipidomics, metabolomics, and glycomics, together with corresponding analyses, may also better aid efforts to understand and control the pathogenesis of ccRCC.

## Conclusions

In summary, a risk model was herein established based on 8 genes found to be significantly associated with TIME composition and prognostic outcomes in ccRCC patients, providing a rational basis for the targeted treatment of this disease on an individualized basis while enabling more effective patient risk stratification. Functional analyses performed based on the risk groups established using this model demonstrated that dysregulated lipid metabolism contributes to impaired immunological activity and fatty acid metabolism reprogramming, resulting in poorer prognostic outcomes.

## Data availability statement

The original contributions presented in the study are included in the article/**Supplementary Materials**, further inquiries can be directed to the corresponding author.

## Ethics statement

The studies involving humans were approved by the Medical Ethics Committee of the Seventh Affiliated Hospital of Sun Yat-sen University. The studies were conducted in accordance with the local legislation and institutional requirements. The human samples used in this study were acquired from primarily isolated as part of your previous study for which ethical approval was obtained. Written informed consent for participation was not required from the participants or the participants’ legal guardians/next of kin in accordance with the national legislation and institutional requirements. The animal study was approved by the Experimental Animal Care Commission of Sun Yat-sen University. The study was conducted in accordance with the local legislation and institutional requirements.

## Author contributions

QZ: Data curation, Formal analysis, Investigation, Methodology, Software, Supervision, Validation, Visualization, Writing - original draft, Writing - review & editing. BL: Data curation, Formal analysis, Investigation, Methodology, Software, Supervision, Validation, Visualization, Writing - original draft. HC: Data curation, Formal analysis, Investigation, Methodology, Software, Supervision, Validation, Visualization, Writing - original draft. YY: Data curation, Formal analysis, Investigation, Methodology, Software, Supervision, Validation, Visualization, Writing - original draft. YH: Data curation, Formal analysis, Investigation, Methodology, Software, Supervision, Validation, Visualization, Writing - original draft. ZC: Data curation, Formal analysis, Investigation, Methodology, Software, Supervision, Validation, Visualization, Writing - original draft. JL: Conceptualization, Data curation, Formal analysis, Funding acquisition, Investigation, Methodology, Project administration, Resources, Software, Supervision, Validation, Visualization, Writing - review & editing.

## Supplementary material

Supplementary Figure 1 Risk model prediction. **(A)** Survival curves demonstrating low- and high-risk patient outcomes for the prediction cohort. **(B)** Risk model ROC curve for the prediction cohort.

Supplementary Figure 2 ALOX5 is over-expressed in human ccRCC specimens. **(A)** The mRNA level expression of ALOX5 in human ccRCC specimens and adjacent normal tissues. **(B)** Representative images of IHC staining of ALOX5 in ccRCC tumor tissues and normal tissues. **(C)** IHC score of ALOX5 in ccRCC tumor tissues and normal tissues. **P<0.01.
