# Supplementary material for: Lipid metabolism-related gene expression in the immune microenvironment predicts prognostic outcomes in renal cell carcinoma
Source: Front Immunol. 2023 Nov 27;14:1324205. doi: 10.3389/fimmu.2023.1324205 (PMC10712371; doi:10.3389/fimmu.2023.1324205)
Supplement: Supplementary Figure 1 — Risk model prediction. (A) Survival curves demonstrating low- and high-risk patient outcomes for the prediction cohort. (B) Risk model ROC curve for the prediction cohort. [file DataSheet_1.zip › Supplementary Data S2.docx]

**Supplementary Data S2**

**Supplementary materials and methods**

Patients and tissue samples

A total of 12 paired human ccRCC specimens and adjacent normal tissues, gathered between 2019 and 2022 from the Department of Urology at the Seventh Affiliated Hospital of Sun Yat-sen University. These samples were promptly frozen in liquid nitrogen upon collection and subsequently stored at -80°C for future analysis. The study received ethical approval from the Medical Ethics Committee of the Seventh Affiliated Hospital of Sun Yat-sen University, and all patients granted informed consent for their participation.

Immunohistochemistry (IHC)

Immunohistochemistry was conducted following established protocols(1). Tissues were fixed in 4% formalin, paraffin-embedded, and 4 μm sections were prepared. After blocking endogenous peroxide activity and nonspecific protein binding, sections were incubated overnight at 4°C with primary antibodies. Subsequently, the sections were washed with PBS and incubated at 37°C for 1 hour with an HRP-polymer-conjugated secondary antibody, followed by DAB staining and hematoxylin counterstaining for 3 minutes(2). Trained investigators, unaware of the experimental conditions, assessed the slides. They quantified the proportions of positively stained cells (0, 0%; 1, 1%‐25%; 2, 26%‐50%; 3, 51%‐75%; 4, 76%‐100%) and staining intensity (0: negative, 1: weak, 2: moderate, 3: strong). The final IHC score was computed by multiplying the intensity score and the percentage score.

References

1. Xiao H, Wang J, Yan W, Cui Y, Chen Z, Gao X, et al. GLUT1 regulates cell glycolysis and proliferation in prostate cancer. Prostate(2018) 78:86-94.doi: 10.1002/pros.23448.
2. Li J, Zhang S, Liao D, Zhang Q, Chen C, Yang X, et al. Overexpression of PFKFB3 promotes cell glycolysis and proliferation in renal cell carcinoma. BMC Cancer(2022) Jan 20;22(1):83. doi: 10.1186/s12885-022-09183-2.

**Figure Legends**

Supplementary Figure S2. ALOX5 is over-expressed in human ccRCC specimens. (A) The mRNA level expression of ALOX5 in human ccRCC specimens and adjacent normal tissues. (B) Representative images of IHC staining of ALOX5 in ccRCC tumor tissues and normal tissues. (C) IHC score of ALOX5 in ccRCC tumor tissues and normal tissues. **P<0.01.
