# Supplementary figures and images for: Lipid metabolism-related gene expression in the immune microenvironment predicts prognostic outcomes in renal cell carcinoma
Source: Front Immunol. 2023 Nov 27;14:1324205. doi: 10.3389/fimmu.2023.1324205 (PMC10712371; doi:10.3389/fimmu.2023.1324205)

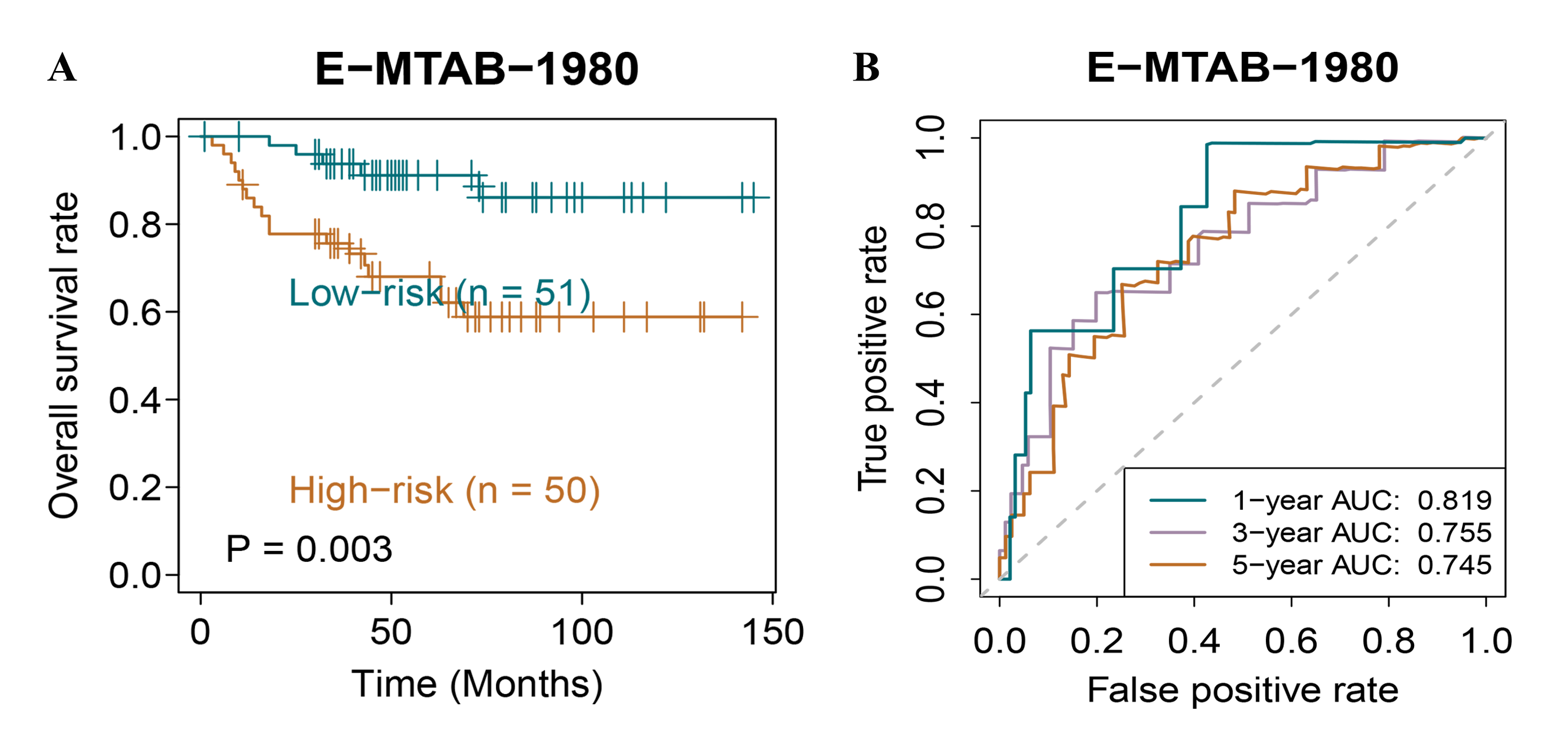

Supplement: Supplementary Figure 1 — Risk model prediction. (A) Survival curves demonstrating low- and high-risk patient outcomes for the prediction cohort. (B) Risk model ROC curve for the prediction cohort. [file DataSheet_1.zip › Supplementary Figure S1.tif]

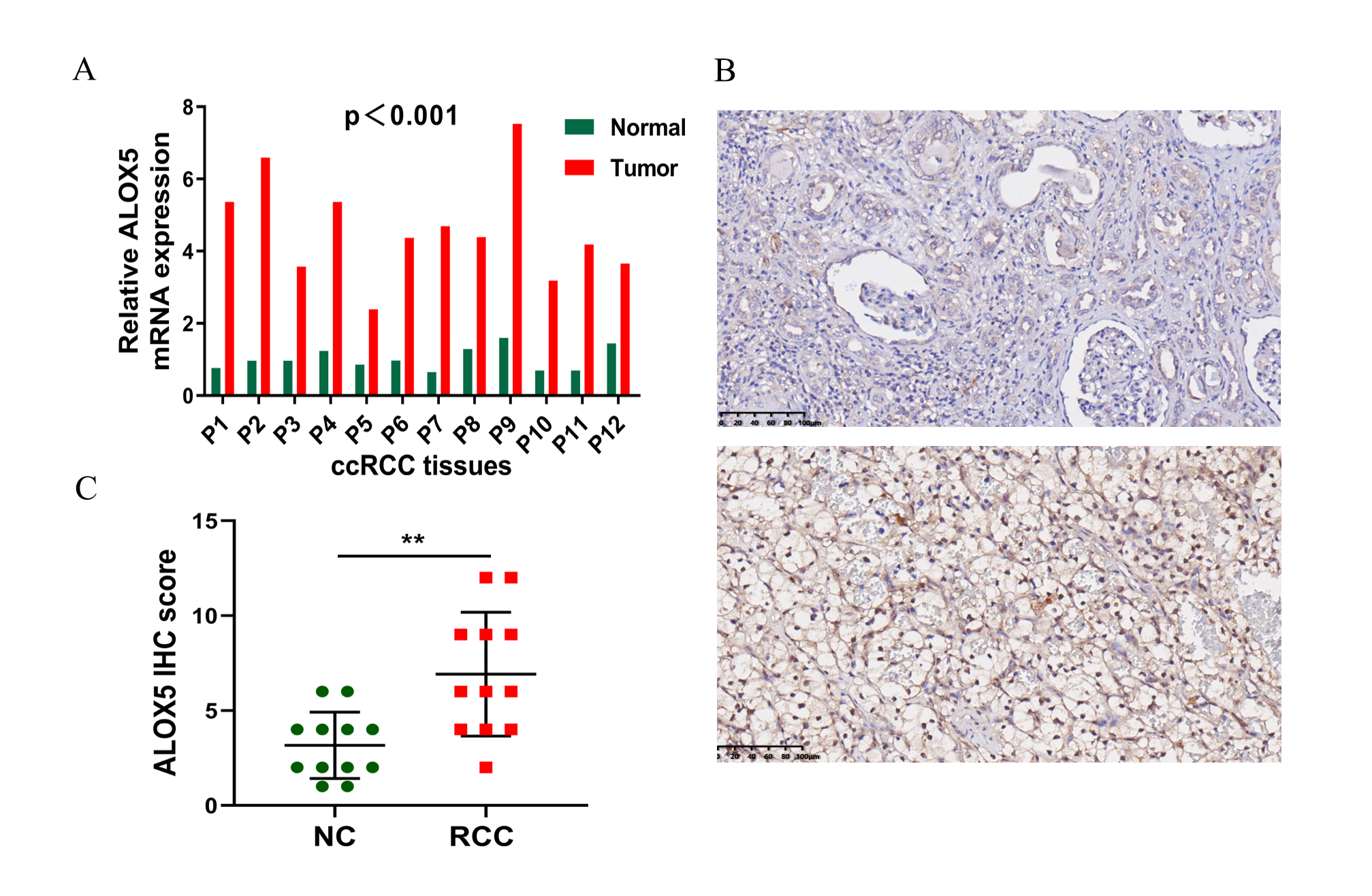

Supplement: Supplementary Figure 1 — Risk model prediction. (A) Survival curves demonstrating low- and high-risk patient outcomes for the prediction cohort. (B) Risk model ROC curve for the prediction cohort. [file DataSheet_1.zip › Supplementary Figure S2.tif]
